# 

**Published:** 1855-09

**Authors:** 


### CONTENTS OF NO. VI, VOL. II.

ORIGINAL COMMUNICATIONS.

Page.

Case of Extreme Narcotism from Opium and treatment—By E C. Atkinson, M. D....................410
The Properties and Uses of Iodine—By W. G. Piper, M. D..............403
Outraged Laws of Hygiene—By Prof. McGugin......................409
On the use of Chloroform in a bite of a Rattle Snake—By Dr. Carpenter.414
Wapello County Medical Society....414
State Medical and Chirurgical Society 417
Suggestions as to the method of using Narcotics in Nervous Diseases—By Prof. Allen....................424

DEPARTMENT OF SELECTIONS.

Nursing Sore Mouth or Puerperal Anæmia—By L. M. Knapp, M. D...429
Meeting of the American Medical Association......................445

EDITORIAL DEPARTMENT.

Extract from Prof. Eve's Valedictory 453
A Quack Remedy for Hydrophobia..459
Virtues of Medical Men ...........461
Dr. Knapp's Treatise.............463
The Iowa Medical Journal.........465
Cincinnati Schools of Medicine......466
Additional Members to the State Medical Society....................467
Chloroform in Fevers..............468

Page

Hot Douche in Typhoid Pneumonia..468
Incontinence of Urine in Children..468
Iodide Potassium ..............468
Rheumatism .....................469
Injections of water and milk in Cholera..........................469
Pyrosis..........................469
Fumes of Opium in Coryza.........469
Hiccough........................469
Iodine and Nitrate of Silver........469
Emmenagogue Properties of Chamomile Flowers....................470
Tinitus Aurum....................471
Nitric Acid in Hooping-cough.... ..471
Leibig's New Broth for the Sick.... 471
Lactate of Iron in Nervous Diseases..472
Solidified Milk...................472
Tincture of Iodine with Chloroform in Inhalation...................472

BIBLIOGRAPHICAL NOTICES.

Clinecal Lectures on Paralysis—By R. B. Todd, M. D., F. R. S......473
Ranking's Abstract...............474
History of American Medical Association—By N. S. Davis, M. D. &c...474
New Hampshire Medical Society.....475
Case of Penetrating Gun-shot Wound of the Heart.................. 477
Tanner's Manual of Clinical Medicine 478
Caution .........................478
Announcements...................479

